# Radiology Review - 6^th^ Edition

**Published:** 2008-02

**Authors:** Darshana A. Sanghvi

**Affiliations:** Department Of Radiology, KEM Hospital and Seth GS Medical College, Mumbai, India. E-mail: darshanasanghvi1@hotmail.com

## Review-1

The 6^th^ edition of Wolfgang Dahnert's *Radiology Review Manual*, like its predecessors, is a good revision guide for exam-going residents as well as a handy reference manual for the practicing general radiologist. The manual essentially provides extensive lists of imaging findings that are associated with a specific pathology as well as lists of differential diagnoses for a given imaging finding. A word of caution: the manual is not meant for the novice or for in-depth reading as there are no detailed conceptual discussions.

The manual is comprehensive. The organization of the text is similar to that of prior editions. The manual is divided into sections covering all the major systems, including MSK, CNS, the orbit, ENT, chest, breast, CVS, abdomen, and obstetrics/gynecology. There is a separate section on nuclear medicine at the end of the book. Additionally, there are two separate chapters on statistics and contrast media. A useful feature is a concise algorithm on the inner front and back covers of the book for treatment of adverse reactions to contrast media. The index at end of the book is thorough and allows easy access to any specific disease entity.

Mnemonics (which are often rather amusing) are liberally found in the book and residents may find them useful for memorizing lists prior to exams. The index includes ‘buzz words’ that have become associated with certain pathologies. Although there are no images in the manual, the line diagrams are lucid and self explanatory. Information is frequently conveyed in the form of easy-to-understand charts and tables.

Noteworthy is the fact that the lists of differential diagnoses are presented in such a manner that statistically common pathologies appear earlier than their rarer counterparts. This feature would be of obvious utility to the student preparing for exams.

The paper and printing in this first Indian reprint are of high quality. Radiologists and radiology residents will find much useful information in this book.

